# Serum uric acid concentration is associated with hypertensive retinopathy in hypertensive chinese adults

**DOI:** 10.1186/s12886-017-0470-y

**Published:** 2017-06-02

**Authors:** Xuling Chen, Ying Meng, Jun Li, Hiacheng She, Liang Zhao, Jing Zhang, Yuan Peng, Kun Shang, Yadi Zhang, Xiaopeng Gu, Wenbin Yang, Yan Zhang, Jianping Li, Xianhui Qin, Binyan Wang, Xiping Xu, Fanfan Hou, Genfu Tang, Rongfeng Liao, Yong Huo, Liu Yang

**Affiliations:** 1 Department of Ophthalmology, Key Laboratory of Vision Loss and Restoration, Ministry of Education, Peking University First Hospital, No.8 Xishiku Street, Xicheng District, Beijing, 100034 China; 2 Department of Tongren Hospital, Beijing, 100005 China; 3 Department of Epidemiology and Biostatistics, School of Public Health, Anhui Medical University, Hefei, Anhui 230032 China; 4 Department of Cardiology, Peking University First Hospital, No.8 Xishiku Street, Xicheng District, Beijing, 100034 China; 5 National Clinical Research Study Center for Kidney Disease; State Key Laboratory for Organ Failure Research; Renal Division, Nanfang Hospital, Southern Medical University, Guangzhou, 510515 China; 6 School of Health Administration, Anhui University, Hefei, 230039 China; 7 Department of Ophthalmology, The First Affiliated Hospital of Anhui Medical University, Hefei, Anhui 230032 China

**Keywords:** Serum Uric Acid Concentration, Hypertensive Retinopathy, Hypertension, Hyperuricemia, Keith-Wagener-Barker system

## Abstract

**Background:**

This cross sectional investigation included 12,966 subjects with hypertension, a cohort of the China Stroke Primary Prevention Trial (CSPPT)**, a** randomized, multicenter clinical trial. This study aimed to explore the correlation between serum uric acid (SUA) concentration and hypertensive retinopathy in hypertensive adults.

**Methods:**

Diagnosis of hypertensive retinopathy was determined by non-mydriatic fundus photography and classified with Keith-Wagener-Barker (KWB) system. The correlation of SUA levels with hypertensive retinopathy prevalence and severity was assessed by statistical analysis.

**Results:**

9848 (75.95%) subjects were diagnosed with hypertensive retinopathy with the following retinopathy grade distribution: grade 1: 58.80%, grade 2: 14.81%, and grade 3–4: 2.34%. SUA levels were significantly associated with hypertensive retinopathy prevalence. Patients with hypertensive retinopathy had higher SUA levels than those without hypertensive retinopathy. Patients in the highest uric acid quartile had an odds ratio for hypertensive retinopathy of 1.21 compared to patients in the lowest uric acid quartile (OR = 1.21, 95% CI: 1.05–1.40, *P* = 0.008). When compared to the non-hyperuricemia group, those in the hyperuricemia group had an odds ratio for hypertensive retinopathy of 1.18(OR = 1.18, 95% CI: 1.05–1.33, *P* = 0.004). Every 1 mg/dl increase in uric acid concentration was significantly associated with a 6% higher odds of hypertensive retinopathy (OR = 1.06, 95% CI: 1.02–1.10, *P* = 0.002).

**Conclusions:**

The prevalence of hypertensive retinopathy was high (75.95%) among hypertensives in our patients cohort. In addition, SUA concentration was significantly associated with hypertensive retinopathy.

## Background

Hypertension affects more than 330 million people in China, it is the most common chronic disease in the Chinese population [1]. Hypertensive retinopathy (HR) is one of the micro vascular complications of hypertension with an insidious onset, which, if left untreated, can possibly open the way for retinal vascular obstruction (RVO), retinal thrombus, ischemic optic neuropathy (ION) and vitreous hemorrhage [2, 3]. Furthermore, the retinal vascular bed, the only vascular bed that can be observed by non-invasive procedures in vivo, is regarded as an essential indicator to evaluate the status of systemic microvasculature [4]. Many studies have proved the value of the retinal vascular in predicting the severity of target organ damage, including cardiovascular, renal and cerebrovascular disease [5, 6]. The prevalence of HR in hypertensive patients varied among different researches (30.6%–94.6%) [7–9]. To date there has been no large sample, epidemiologic statistical reports on HR in China.

Some studies have explored the risk factors for HR, including endothelial dysfunction [10], oxidative stress [11], and low-grade systemic inflammation [12]. However, other risk factors, such as serum triglyceride levels, serum uric acid (SUA) and metabolic syndrome, have been studied with relatively inconclusive results. Uric acid is the final product of purine metabolism in humans. Hyperuricemia is a predisposing condition for gout and is linked with metabolic syndrome [13], resulting from increased production of uric acid coupled with excretion dysfunction. It was also regarded as an independent risk factor for hypertension. Two meta-analysis of published prospective studies showed that the overall risk for incident hypertension increased by 13% and 15% per 1 mg/dl increase in SUA level respectively [14, 15]. Numerous studies have suggested that SUA is correlated with some ocular diseases [16], especially diabetic retinopathy [17]. Our study sought to characterize epidemiological features of HR and investigate the association between SUA and HR. We were interested whether SUA is an independent risk factor contributing to HR.

## Methods

### Study design and participants

All subjects in this study came from the China Stroke Primary Prevention Trial (CSPPT), conducted from May 19, 2008, to August 24, 2013, in 32 communities in the Jiangsu and Anhui provinces of China [18]. The study complied with the Helsinki Declaration and was approved by the Ethics Committee of the Institute of Biomedicine, Anhui Medical University, Hefei, China (FWA assurance number FWA00001263). All participants provided written informed consent. CSPPT study was a large community-based, randomized, multicenter, double blind, and actively controlled trial designed to evaluate whether combination therapy with enalapril maleate and folic acid tablets combined were more effective in preventing stroke in Chinese adults with hypertension than enalapril maleate alone. Details of the trial have been described elsewhere (http://clinicaltrials.gov/ct2/show/NCT00794885).

Our study included a total of 20,702 hypertensive subjects, 13,140 had fundus picture, among them, and 155 subjects were excluded for difficult gradable fundus photographs, for the reason of serious opacity of refractive media or terrible fixation vision. After excluding 7736 subjects who were missing either gradable fundus photographs or analysis of SUA, a total of 12,966 subjects were analyzed.

### Classification of Hypertensive Retinopathy

Non-mydriatic fundus photographs were taken in the posterior pole and macula-centered, using fundus cameras (Topcon TRC-NW8 Non-Mydriatic Retinal Camera, Canon CR-2 AF Non-Mydriatic Retinal Camera and Kowa nonmyd 7 Fundus Camera). All the photographs were randomly evaluated by four professional ophthalmologists with double mask, and we ensured the results of science and reliability through good consistency checks (kappa between 0.71–0.95). HR was classified into grades 1–4 according to the Keith-Wagener-Barker (KWB) system (for details of classification see [19] Table 1).

**Table 1:** The Keith–Wagener–Barker classification system for hypertensive retinopathy

Grade	Features
None	No detectable positive signs
1	Mild or moderate generalized retinal arteriolar narrowing, arteriovenous tortuosity
2	Definite focal narrowing and arteriovenous nipping, crossing compression
3	Copper wire or silver wire artery, signs of grade 2 retinopathy plus retinal hemorrhages, exudates and cotton wool spots
4	Severe grade 3 retinopathy plus papilledema or retinal edema

### Laboratory Examinations

Laboratory examinations were performed at the core lab of the National Clinical Research Center for Kidney Disease (Nanfang Hospital, Guangzhou, China). Fasting serum uric acid (SUA), lipids (serum total cholesterol, high density lipoprotein – HDL-C, and triglycerides), serum creatinine, blood glucose and homocysteine were measured by using automatic clinical analyzers (Beckman Coulter), serum folic acid were measured by using a chemiluminescent immunoassay (New Industrial). Diabetes mellitus (DM) was defined as a fasting plasma glucose concentration greater than or equal to 7.0 mmol/l, or a history of diabetes paired with the use of an oral anti-diabetic drug. Hyperuricemia was defined as serum uric acid exceeding 7.0 mg/dl in males and 6.0 mg/dl in females.

### Demographic data

All participants were interviewed using a standardized questionnaire including age, gender, sociodemographic status, education, occupation, diet, lifestyle, health behavior, medical history and personal history including smoking status, alcohol consumption, and known systemic disease. Current smoking was defined as smoking one cigarette per day for at least half a year. Current drinking was defined as drinking once per week for at least half a year. Anthropometric measurements were taken according to a standard operating procedure. Body Mass Index (BMI) was calculated as weight (kilograms) divided by height (meters) squared.

### Blood Pressure (BP) measurements

Systolic and diastolic BP (SBP/DBP mmHg) was measured after subjects resting for 30 min; participants were seated with their right arms supported at the level of the heart for BP measurements. BP was measured using a mercury sphygmomanometer with an appropriate cuff size, and recorded as the mean of three measurements, with one-minute intervals between each. Hypertension was defined as a BP greater than or equal to 140 mmHg systolic and/or 90 mmHg diastolic.

### Statistical analysis

All analyses were performed using Empower Stats statistical software (http://www.empowerstats.com, X&Y Solutions, Inc. Boston, MA) and the statistical package R(http://www.r-project.org). Data were presented as frequencies (percentages) for categorical variables and means ± standard deviation (SD) for continuous variables. Stratified analysis, interaction tests, and covariate screening were performed. Binary logistic regression analyses were used to assess the associations between HR (as a binary variable) and SUA concentration. SUA was evaluated as a continuous variable, a bi-level classification variable (Non-hyperuricemia: SUA <7.0 mg/dl in males, SUA < 6.0 mg/dl in females; Hyperuricemia: SUA ≥7.0 mg/dl in males, SUA ≥ 6.0 mg/dl in females) and as quartiles (quartile 1: <4.4 mg/dl; quartile 2: 4.4–5.3 mg/dl; quartile 3: 5.3–6.4 mg/dl; quartile 4: ≥6.5 mg/dl). The results were shown as odd ratios (OR) and 95% confidence intervals (CI) with adjustment for major variables including age, sex, study center, treatment group, body mass index (BMI), SBP and DBP, triglycerides, fasting plasma glucose, creatinine. A two-tailed *P* value of <0.05 was considered statistically significant.

## Results

The present study included 12,966 subjects(mean age: 63.9 ± 7.3 years, range 44–81 years, 5206 males and 7760 females, data not shown). Among them, 9848 (75.9%) had HR, the prevalence in males and females was 77.91% and 74.64%, respectively. Grade 1 retinopathy was diagnosed in 58.80% (*n* = 7624), grade 2 in 14.81% (*n* = 1921), and grade 3 in 2.34% (*n* = 303). Only three subjects were diagnosed as HR grade 4, so these grade 4 subjects were merged with the grade 3 group for analysis.

Demographic and anthropometric characteristics and the laboratory results of the subjects are listed in Table 2. Subjects were divided into two groups: those without HR, the non-HR (NHR) group and those with HR, the HR group. There were significant differences in gender distribution (*P* < 0.001), systolic blood pressure (SBP; *P* < 0.001), diastolic blood pressure (DBP; *P* < 0.001), SUA (*P* < 0.001), folic acid (*P* = 0.044), creatinine (*P* < 0.001), study center location (*P* < 0.001) and consumption of alcohol (*P* = 0.001) in the two groups. Compared to NHR group, HR group had higher SBP, DBP, SUA, and creatinine levels. The mean value of SUA was 5.56 mg/dl and 5.36 mg/dl for the HR and NHR groups, respectively (Table 2).

**Table 2:** Baseline characteristics of the study participants

	Total	NHR group	HR group	*P* value
Characteristics	(*n* = 12,966)	(*n* = 3118, 24.05%)	(*n* = 9848, 75.95%)	
Age (years)	63.90 ± 7.30	63.70 ± 7.30	63.96 ± 7.30	0.085
Gender				<0.001
Male	5206 (40.20%)	1150 (36.90%)	4056 (41.20%)	
Female	7760 (59.80%)	1968 (63.10%)	5792 (58.80%)	
BMI (kg/m^2^)^a^	25.10 ± 3.81	25.01 ± 3.78	25.14 ± 3.82	0.105
SBP (mmHg)	135.80 ± 17.48	134.81 ± 16.88	136.11 ± 17.65	<0.001
DBP(mmHg)	82.25 ± 10.89	81.27 ± 10.49	82.56 ± 10.99	<0.001
Uric Acid (mg/dl)	5.51 ± 1.49	5.36 ± 1.42	5.56 ± 1.51	<0.001
Creatinine (μmol/l)	68.09 ± 25.86	66.39 ± 21.99	68.63 ± 26.95	<0.001
Triglycerides (mmol/l)	1.80 ± 1.43	1.76 ± 1.34	1.81 ± 1.45	0.086
Glucose (mmol/l)	6.27 ± 2.03	6.24 ± 1.87	6.29 ± 2.07	0.261
Folic Acid (ng/ml)	18.46 ± 14.70	17.99 ± 13.37	18.61 ± 15.09	0.044
DM				0.14
No	10,089 (80.70%)	2466 (81.60%)	7623 (80.40%)	
Yes	2412 (19.30%)	555 (18.40%)	1857 (19.60%)	
Study Center			<0.001	
Anqing	2319 (17.90%)	420 (13.50%)	1899 (19.30%)	
Lianyungang	10,647 (82.10%)	2698 (86.50%)	7949 (80.70%)	
Treatment Group			0.985	
Enalapril only	6531 (50.40%)	1571 (50.40%)	4960 (50.40%)	
Enalapril-Folic Acid	6435 (49.60%)	1547 (49.60%)	4888 (49.60%)	
Smoker				0.15
Never	8697 (67.60%)	2135 (69.00%)	6562 (67.20%)	
Former	1404 (10.90%)	316 (10.20%)	1088 (11.10%)	
Current	2756 (21.40%)	643 (20.80%)	2113 (21.60%)	
Alcohol Consumption				0.001
Never	8733 (70.80%)	2184 (73.40%)	6549 (69.90%)	
Former	838 (6.80%)	176 (5.90%)	662 (7.10%)	
Current	2770 (22.40%)	615 (20.70%)	2155 (23.00%)	

*Abbreviations*: *SBP* Systolic Blood Pressure, *DBP* Diastolic Blood ressure, *DM* diabetes mellitus, *Treatment Group* Enalapril only, Enalapril-Folic Acid, *BMI* body mass index

*NHR group* non-hypertensive retinopathy, *HR group* hypertensive retinopathy

^a^Calculated as weight in kilograms divided by height in meters squared

SUA levels ranged from 1.70 to 16.23 mg/dl among all participants. Subjects were stratified into four groups according to their SUA quartiles. The percentage of HR prevalence was 73.38% for subjects in the first SUA quartile (<4.4 mg/dl), 74.26% in the second SUA quartile (4.4–5.3 mg/dl), 76.65% in the third SUA quartile (5.3–6.4 mg/dl), and 79.47% in the fourth SUA quartile (≥6.5 mg/dl; Table 3). A positive trend was observed, with increased SUA quartiles, the prevalence of HR significantly increased (*P* < 0.001). To determine any difference in trend between men and women, data were stratified by gender. As shown in Table 3, male subjects had a higher prevalence of HR than females in all SUA quartiles, thus the association between SUA and HR remained unaltered. Subjects were further divided into a non-hyperuricemia group (*n* = 10,007) and a hyperuricemia group (*n* = 2959) according to clinical SUA values defined previously. The hyperuricemia group had a higher prevalence of HR (79.48%) than the non-hyperuricemia group (74.97%). Collectively, these data suggested that increased SUA levels were associated with an increased prevalence of HR.

**Table 3:** Prevalence of HR and the association between HR and SUA

		Prevalence of HR (%)						
Serum Uric Acid (mg/dl)	*N*	Total	Males	Females	Model 1		Model 2	
					OR, 95% CI	*P*	OR, 95% CI	*P*
Continuous variable	12,966	75.95	77.90	74.60	1.10 (1.07, 1.13)	<0.001	1.06 (1.02, 1.10)	0.002
Quartiles								
Q1 (<4.4)	3201	73.38	76.73	72.75	1		1	
Q2 (4.4–5.3)	3248	74.26	75.95	73.51	1.05 (0.936, 1.17)	0.423	1.00 (0.891, 1.13)	0.982
Q3 (5.3–6.4)	3259	76.65	77.22	76.15	1.19 (1.06, 1.33)	0.002	1.12 (0.988, 1.27)	0.076
Q4 (≥6.5)	3258	79.47	79.62	79.16	1.40 (1.25, 1.58)	<0.001	1.21 (1.05, 1.40)	0.008
Binary Classification								
Non-hyperuricemia	10,007	74.97	77.00	73.60	1		1	
Hyperuricemia	2959	79.48	80.40	78.50	1.28 (1.16, 1.42)	<0.001	1.18 (1.05, 1.33)	0.004

*Non-hyperuricemia* SUA < 7.0 mg/dl in males and SUA < 6.0 mg/dl in females, *hyperuricemia* SUA ≥7.0 mg/dl in males and SUA ≥6.0 mg/dl in females

Binary logistic regression models evaluating the association of serum uric acid (SUA) with hypertensive retinopathy (HR). *CI* confidence interval *OR* odds ratio

Model 1: unadjusted

Model 2: adjusted for age, sex, study center, treatment group, body mass index(BMI), SBP, DBP, creatinine, triglycerides, and fasting plasma glucose if not stratified

In order to accurately determine the relationship between SUA and HR, we conducted stratified analyses, interaction tests, and covariate screening. The screening criteria included any risk factor that produced a > 10% change in the regression coefficient after introduction into the basic model. The results show that triglyceride levels, creatinine levels, SBP, DBP, age, BMI, study center, and gender met the filter criteria. Binary logistic regression models were run after adjusting for confounding variables (gender, age, treatment group, study center, DM, BMI, creatinine levels, SBP, and DBP). The results showed that SUA remained significantly associated with HR (OR = 1.06, 95% CI: 1.02–1.10, *P* = 0.002), indicating that every 1 mg/dl increase in SUA concentration is associated with a 6% higher odds of retinopathy after adjusting for multiple confounders. For patients with SUA in the fourth quartile (≥6.5 mg/dl), a 1 mg/dl increase in SUA was significantly associated with a 21% higher odds of retinopathy (OR = 1.21, 95% CI: 1.05–1.40, *P* = 0.008) after adjusting for multiple confounders, when compared to patients with SUA in the 1st quartile (<4.4 mg/dl). As a binary variable, compared with the non-hyperuricemia group, the OR for the hyperuricemia group was 1.18 (95% CI: 1.05–1.33, *P* = 0.004).

**Fig. 1:**
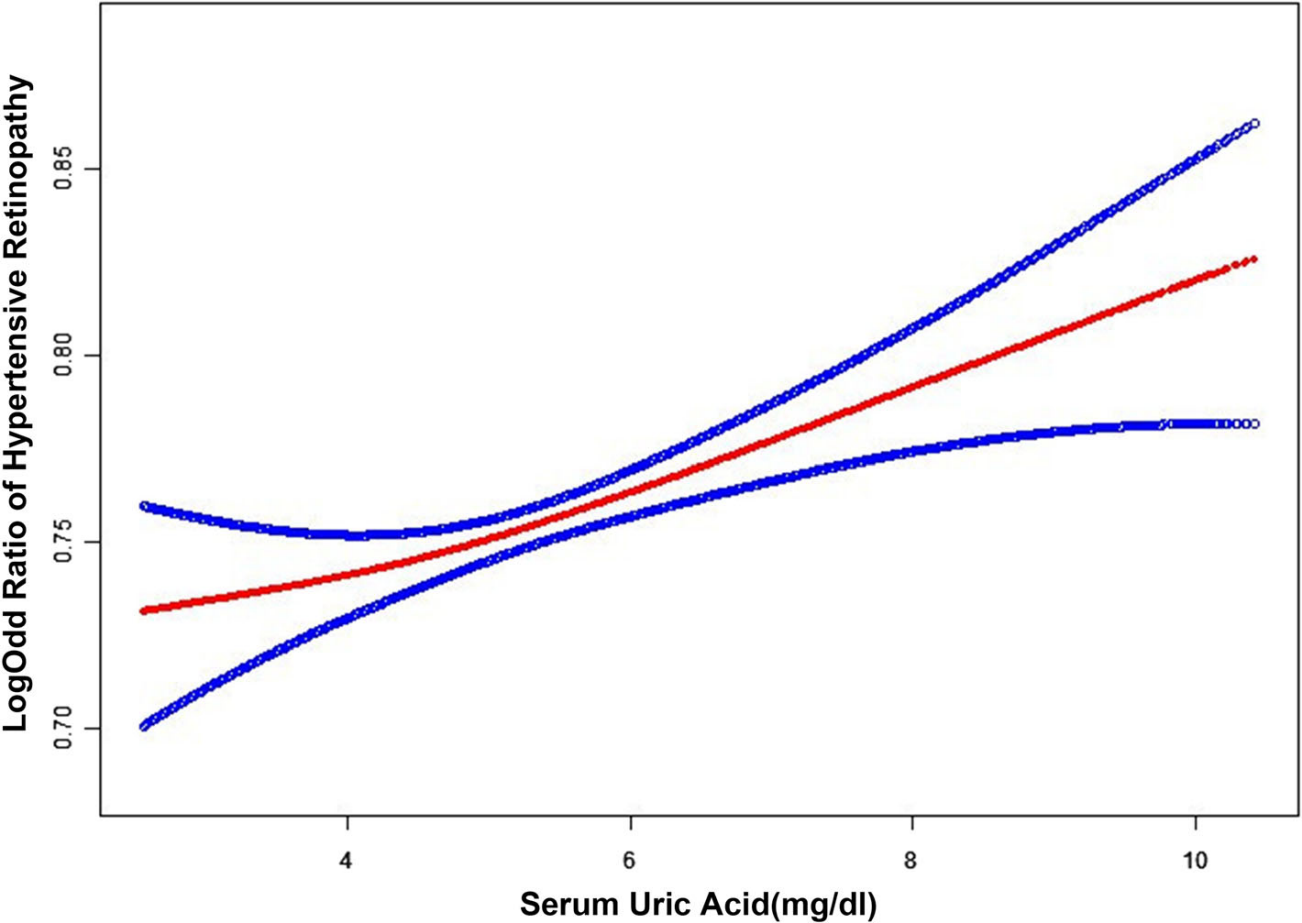
Smooth curve fitting of hypertensive retinopathy and serum uric acid. Data from 0.5% of the tails on either end of the distribution were deleted prior to performing a smooth curve fitting after adjusting for all variables. With increasing serum uric acid (SUA) levels, the risk of hypertensive retinopathy (HR) increases. The *red line* represents the best-fit line; the *blue lines* are 95% confidence intervals

Data from 0.5% of the tails on either end of the distribution were deleted prior to performing a smooth curve fitting after adjusting for all variables. Figure 1 showed the resultant curve indicating that with increasing SUA levels, the risk of HR increases. We further explored the relationship between HR and SUA through stratified analysis where SUA was modeled as a continuous variable (Table 4). Interaction tests were performed to determine any impact of each stratified variable on the relationship between SUA and HR. (P ⩽0.05 was considered significant). No significant effect on SUA or HR was observed. The results indicated that there were no confounding factors between the variables. For women, a 1 mg/dl increase in SUA concentration was associated with a 9% higher odds of retinopathy (OR = 1.09, 95% CI: 1.04–1.14, *P* < 0.001). For those under 60 years of age, a 1 mg/dl increase in SUA concentration was associated with an 11% higher odds of retinopathy (OR = 1.11, 95% CI: 1.04–1.18, *P* = 0.002). For Non-DM, a 1 mg/dl increase in SUA concentration was associated with an 5% higher odds of retinopathy (OR = 1.05, 95% CI: 1.01–1.09, *P* = 0.028) (Table 4).

**Table 4:** Stratified analysis of the association of SUA on the risk of hypertensive retinopathy (SUA as a continuous variable)

	*n*	Events	%	Model 1		Model 2		*P* value for interaction
				OR (95% CI)	*P*	OR (95% CI)	*P*	
Gender								0.122
Male	5206	4056	77.91	1.07 (1.02, 1.11)	0.005	1.02 (0.96, 1.07)	0.593	
Female	7760	5792	74.64	1.10 (1.06, 1.15)	<0.001	1.09 (1.04, 1.14)	<0.001	
Age (years)								0.129
< 60	8846	6759	76.41	1.14 (1.08, 1.20)	<0.001	1.11 (1.04, 1.18)	0.002	
≥ 60	4120	3089	74.98	1.08 (1.04, 1.11)	<0.001	1.03 (0.99, 1.08)	0.154	
Treatment Group								0.267
Enalapril only	6531	4960	75.95	1.08 (1.04, 1.12)	<0.001	1.05 (1.00, 1.10)	0.076	
Enalapril-Folic Acid	6435	4888	75.96	1.11 (1.07, 1.16)	<0.001	1.07 (1.02, 1.13)	0.010	
SBP (mmHg)								0.535
< 127.0	4163	3088	74.18	1.11 (1.06, 1.16)	<0.001	1.08 (1.01, 1.14)	0.019	
127.0 ≤ SBP < 141.3	4204	3197	76.05	1.12 (1.07, 1.18)	<0.001	1.09 (1.03, 1.16)	0.006	
≥ 141.3	4448	3447	77.5	1.07 (1.02, 1.12)	0.008	1.01 (0.95, 1.07)	0.846	
DBP (mmHg)								0.083
< 78.0	4145	3071	74.09	1.10 (1.05, 1.15)	<0.001	1.06 (1.00, 1.12)	0.069	
≥ 78.0, <86.5	4392	3303	75.2	1.14 (1.08, 1.20)	<0.001	1.09 (1.03, 1.16)	0.005	
≥ 86.5	4278	3358	78.49	1.05 (0.99, 1.10)	0.083	1.02 (0.95, 1.08)	0.618	
DM								0.834
No	10,089	7623	75.56	1.10 (1.07, 1.14)	<0.001	1.05 (1.01, 1.09)	0.028	
Yes	2412	1857	76.99	1.09 (1.02, 1.15)	0.009	1.07 (0.99, 1.16)	0.077	

*Abbreviations*: *SBP* Systolic Blood Pressure, *DBP* Diastolic Blood Pressure, *DM* diabetes mellitus, *Treatment Group* Enalapril only, Enalapril-Folic Acid

Binary logistic regression models evaluating the association of serum uric acid(SUA) with hypertensive retinopathy(HR). *CI* confidence interval, *OR* odds ratio

Model 1: unadjusted

Model 2: adjusted for age, sex, study center, treatment group, body mass index(BMI), SBP, DBP, creatinine, triglycerides, and fasting plasma glucose if not stratified

Table 5 shows the results of SUA modeled in quartiles and as a binary variable. We observed a progressive and significant increase in the odds of retinopathy with SUA levels in the fourth quartile for females (OR = 1.35, 95% CI: 1.12–1.63, *P* = 0.002) and in the enalapril-folic acid treatment group (OR = 1.29, 95% CI: 1.05–1.58, *P* = 0.016). A similar trend was also seen in the second quartile of SBP and DBP. When compared with the non-hyperuricemia group, a 1 mg/dl increase in SUA concentration was associated with a 26% higher odds of retinopathy in females with hyperuricemia (OR = 1.26, 95% CI: 1.09–1.46, *P* = 0.002) and a 38% higher odds of retinopathy in those under 60 with hyperuricemia (OR = 1.38, 95% CI:1.12–1.70, *P* = 0.003). Similarly, this trend was observed in the enalapril folic acid treatment group, the second quartile of SBP, the first and second quartile of DBP, and in patients without DM (Table 5).

**Table 5:** Stratified analysis of the association of SUA on the risk of hypertensive retinopathy (SUA quartiles and as binary variable)

Variable	SUAmg/dl (OR, 95% CI, *P* value)							
	Q1	Q2	Q3	Q4	*P* value for interaction	Non-hyperuricemia	Hyperuricemia	*P* value for interaction
Gender					0.297			0.235
Male	1	0.93 (0.72, 1.22) 0.616	0.96 (0.74, 1.23) 0.721	1.00 (0.77, 1.29) 0.970		1	1.06 (0.88, 1.27) 0.531	
Female	1	1.01 (0.89, 1.15) 0.877	1.19 (1.03, 1.38) 0.022	1.35 (1.12, 1.63) 0.002		1	1.26 (1.09, 1.46) 0.002	
Age (years)					0.026			0.149
< 60	1	1.10 (0.95, 1.27) 0.197	1.12 (0.96, 1.31) 0.149	1.19 (1.00, 1.41) 0.054		1	1.38 (1.12, 1.70) 0.003	
≥ 60	1	0.84 (0.70, 1.02) 0.084	1.12 (0.91, 1.39) 0.277	1.28 (1.00, 1.65) 0.054		1	1.09 (0.95, 1.26) 0.197	
Treatment Group					0.302			0.0301
Enalapril only	1	1.04 (0.89, 1.23) 0.603	1.12 (0.94, 1.33) 0.215	1.15 (0.94, 1.40) 0.172		1	1.05 (0.90, 1.23) 0.509	
Enalapril-Folic Acid	1	0.96 (0.81, 1.13) 0.604	1.12 (0.94, 1.34) 0.198	1.29 (1.05, 1.58) 0.016		1	1.34 (1.13, 1.58) <0.001	
SBP (mmHg)					0.671			0.165
< 127.0	1	1.04 (0.85, 1.28) 0.715	1.19 (0.96, 1.48) 0.107	1.21 (0.95, 1.54) 0.128		1	1.20 (0.99, 1.46) 0.070	
≥ 127.0, <141.3	1	0.93 (0.76, 1.14) 0.469	1.08 (0.87, 1.33) 0.499	1.38 (1.07, 1.77) 0.013		1	1.35 (1.10, 1.66) 0.004	
≥ 141.3	1	1.04 (0.85, 1.26) 0.731	1.09 (0.88, 1.35) 0.449	1.08 (0.85, 1.38) 0.533		1	1.03 (0.85, 1.25) 0.787	
DBP (mmHg)					0.326			0.182
< 78.0	1	0.99 (0.81, 1.20) 0.893	1.17 (0.95, 1.46) 0.143	1.10 (0.86, 1.39) 0.458		1	1.22 (1.00, 1.49) 0.047	
≥ 78.0, <86.5	1	1.06 (0.88, 1.29) 0.523	1.17 (0.95, 1.43) 0.141	1.43 (1.12, 1.83) 0.004		1	1.23 (1.01, 1.51) 0.039	
≥ 86.5	1	0.92 (0.74, 1.15) 0.473	0.99 (0.79, 1.24) 0.902	1.09 (0.85, 1.41) 0.485		1	1.08 (0.89, 1.32) 0.445	
DM					0.998			0.941
No	1	0.98 (0.86, 1.11) 0.732	1.10 (0.96, 1.27) 0.181	1.15 (0.98, 1.35) 0.097		1	1.16 (1.02, 1.32) 0.028	
Yes	1	1.05 (0.80, 1.38) 0.716	1.20 (0.90, 1.60) 0.209	1.34 (0.97, 1.85) 0.073		1	1.21 (0.93, 1.57) 0.148	

*Abbreviations*: *SBP* Systolic Blood Pressure, *DBP* Diastolic Blood Pressure, *CI* confidence interval, *OR* odds ratio

Q1: <4.4 mg/dl; Q2: 4.4–5.3 mg/dl; Q3: 5.3–6.4 mg/dl; Q4: ≥6.5 mg/dl; DM,diabetes mellitus; Treatment Group: Enalapril only, Enalapril-Folic Acid

Non-hyperuricemia: SUA <7.0 mg/dl in males and SUA <6.0 mg/dl in females; hyperuricemia: SUA ≥7.0 mg/dl in males and SUA ≥6.0 mg/dl in females

## Discussion

Our study was the first community-based epidemiologic study of HR in rural China and provided valuable information on the epidemiology of HR among a hypertensive population. The prevalence of HR in our study was 75.95%, much higher than that reported in studies from Korea (61.3%), and India (30.6%) [8]. This may potentially reflected the higher incidence of hypertension in rural China and less concern and/or lack of knowledge of the complications from hypertension. Identification of clinical features that could be used to predict the development and progression of retinopathy is of crucial importance for hypertensive patients.

In recent years, numerous studies have indicated a relationship between uric acid and ocular disease. Some ocular manifestations of gout, which is characterized by a rise in SUA, have been reported, including corneal uric acid crystals, band keratopathy, increased intraocular pressure, asteroid hyalosis, conjunctival injection, and uveitis [20]. Patients with normal-tension glaucoma (NTG) have higher SUA levels than controls [21]. Retinal vein occlusion (RVO) in both eyes often accompanied with hyperuricemia [22]. In addition, uric acid also played an important role in diabetic retinopathy (DR). A 3-year prospective study in patients with Type 2 DM indicated that SUA concentration was associated with an increase in the severity of DR [23]. SUA may also be involved in the pathogenesis and progression of DR [24]. Researches all above presented the negative influence on eyes. However, some studies maintained that the effect of uric acid was that of an antioxidant, may protect the retina from oxidative damage. Morsal et al. [25] supported the concept that elevated SUA levels may provide a therapeutic approach for the treatment of ARMD. Several studies have established important relationships between low uric acid and neuromyelitis optica [26, 27]. Low SUA appeared to precede the incidence of DR, and that SUA declined further as the disease progresses [28]. According to previous literature, it was observed that although the association between HR and SUA was somehow inconsistent, most results of researches proved more strong evidence that SUA as a destructive factor and aggravated ocular disease, including normal-tension glaucoma (NTG), retinal vein occlusion (RVO) and diabetic retinopathy (DR) [21–24]. In our study, SUA concentration was significantly associated with HR; the results supported the adverse effect of uric acid on HR.

The prevalence of hyperuricemia in hypertensive patients was reported between around 20%–50% [29, 30]. Significant epidemiological evidence showed that uric acid might be associated with hypertension [31]. An elevation in SUA was associated with an increased risk for the development of hypertension [32]. Therefore, we hypothesized uric acid tend to play as a provident in pathogenesis of HR and promote its development and progression.

The role of uric acid as a mediator of vascular damage is not a new idea but has only recently gained widespread acceptance [29]. Increased levels of SUA can lead to endothelial cell dysfunction, which is an important step in the development of atherosclerosis, via nitric oxide synthase and stimulation of vascular smooth muscle cell proliferation [32]. Rat models of hyperuricemia suggest that hyperuricemia leads to hypertension in a stepwise fashion. In the first phase, uric acid–dependent activation of the renin–angiotensin system and down regulation of nitric oxide (NO) production, led to vasoconstriction. At this stage, uric acid reduction results in vascular relaxation and improved blood pressure. The second phase, which develops over time, is characterized by uric acid–mediated arteriolosclerosis. This process is not reversed by later reduction of uric acid, and causes permanent sodium-sensitive hypertension [33]. Retinal vasculature and other target organs share similar anatomical features and physiological properties. The effect of SUA on the systemic blood vessels also affects the retinal vascular bed. HR can be divided into four stages according to physiology, vasoconstriction, sclerosis, exudation and complications [34]. High blood pressure stimulates the constriction of vessels, and long-term vasoconstriction causes vasospasm and vascular endothelium hypoxia-ischemia. These processes culminate in vascular smooth muscle cell proliferation, vascular wall thickening and vessel stenosis [35]. Uric acid may have a similar effect as high blood pressure on vessels and prompts the vascular sclerosis in union.

We observed interesting phenomena that SUA may have a greater influence in women and non-DM. Compared to men, women have lower SUA levels and fewer cardiovascular risk factors [36]. Much experimental and epidemiological evidence suggested that estrogen exert a vascular protective function and may have beneficial effects on endothelial function and atherosclerosis, raising the possibility of sex differences in arterial remodeling [37]. However, many studies indicated that SUA had greater influence on women [38, 39]. Recent studies showed a relationship between SUA and coronary atherosclerosis in women only [40, 41]. In patients with hyperuricemia, women have higher risk of left ventricular hypertrophy than men [42]. Similar phenomenon also appeared in our study, for women, a 1 mg/dl increase in SUA concentration was associated with a 9% higher odds of retinopathy, while the corresponding values were not significant for men. The hormonal changes may be the key point. Hormonal changes after menopause increased the risk for cardiovascular diseases [43]. Sudden withdrawal of estrogen affected developing endothelial dysfunction in menopause [44]. In our study, subjects were between 44 and 81 years old. Women in this study had either reached perimenopause, were in menopause or were post-menopausal, and all had reduced protective effects of estrogen, making them more vulnerable to oxidants. This may be a possible explanation for the significant association between SUA and HR in women in our cohort. In addition, Iemolo et al. studied sex differences in carotid plaque and stenosis, and concluded that women have more stenosis but fewer plaques than men, suggesting that differences in sex hormones may affect remodeling of atherosclerosis. So we inferred that there was another possibility that women may display significant differences in the appearance of HR. Generally, in the evaluation of HR, stenosis was emphasized rather than atherosclerosis, which may partly explain why women have a higher rate of HR.

For the non-DM group, a 1 mg/dl increase in SUA concentration was associated with a 5% higher odds of retinopathy while the odds were not significantly changed in the DM group. This result seemed to contradict previous studies, indicating that DR was associated with SUA. Uric acid, high blood pressure and high blood glucose all have destructive effect on vessels [45, 46]. Typical early retinopathy signs of DM and hypertension share many morphological and pathophysiological similarities [47]. We concluded that glucose may play a more important role in the DM group in our cohort than uric acid, and the subjects in the non-DM group were more sensitive to uric acid.

Our study was limited in the following aspects. First, the CSPPT participants were rural Chinese, thus the generalizability of our findings to other populations requires caution. Second, more than 30% were excluded because of the lack of photographic material or SUA analysis, this group may have potential influence on our result, however, we thought this group did not differed from the total population in terms of the main variables of interest so the influence was minor. Third, there were a limited number of subjects with grade 3 and grade 4 HR. Fourth, there were lacks of assessments on other hypertension related ocular diseases, which may also be affected by SUA. Fifth, the non-mydriatic fundus photography was imperfect. Due to the study was a large community-based trial, we were not allowed to do dilated eye examination to every person. Hypertension mainly affected retinal arteries and their first or second branches, and it caused lesions distributed peripapillary, which can be covered by non-mydriatic fundus photographs mostly, although we may not be able to catch changes of peripheral fundus. Many other studies about HR also adopted non-mydriatic retinal photograph or performed direct ophthalmoscopy without mydriasis [6, 48]. Besides, fundus examination was not free from subjectivity, and the KWB classification system was still controversial. Finally, the study lacked any long-term follow up investigation into the impact of uric acid. In order to provide an in-depth examination of the relationship between SUA and HR, a longer term follow-up study including more detailed factors is required.

## Conclusion

In conclusion, this cross-sectional study demonstrated that SUA concentration was positively associated with odds of HR, every 1 mg/dl increase in SUA concentration was associated with a 6% higher odds of retinopathy. We emphasized the effect of metabolic factors on HR and speculated that SUA provided useful information to physicians for the timing of funduscopic examinations in hypertensive. Further elucidation of the underlying mechanism that SUA promotes HR may need further study. Whether SUA lowing can reduce a risk of HR still need deeper discussion and clinical trials.
